# DC120, a novel AKT inhibitor, preferentially suppresses nasopharyngeal carcinoma cancer stem-like cells by downregulating Sox2

**DOI:** 10.18632/oncotarget.3128

**Published:** 2015-02-04

**Authors:** Juan Qin, Jiao Ji, Rong Deng, Jun Tang, Fen Yang, Gong-Kan Feng, Wen-Dan Chen, Xiao-Qi Wu, Xiao-Jun Qian, Ke Ding, Xiao-Feng Zhu

**Affiliations:** ^1^ State Key Laboratory of Oncology in South China, Cancer Center, Sun Yat-sen University, Guangzhou, China; ^2^ Department of Breast Oncology, Sun Yat-sen University, Guangzhou, China; ^3^ Graduate School of Chinese Academy of Sciences, Beijing, China; ^4^ Key Laboratory of Regenerative Biology and Institute of Chemical Biology, Guangzhou Institutes of Biomedicine and Health, Chinese Academy of Sciences, Guangzhou China

**Keywords:** DC120, PKB/AKT, nasopharyngeal carcinoma, cancer stem-like cells

## Abstract

Side population (SP) contains cancer stem-like cells (CSLCs). In this study, we characterized SP cells from nasopharyngeal carcinoma (NPC) cell lines and found that SP cells had a higher self-renewal ability *in vitro* and greater tumorigenicity *in vivo*. The AKT pathway was activated in NPC SP cells. DC120, a 2-pyrimidyl-5-amidothiazole inhibitor of the ATP binding site of AKT, inhibited phosphorylation of FKHRL1 and GSK-3β. DC120 inhibited SP fraction, the sphere-forming ability *in vitro* and growth of primary xenografts as well as secondary xenografts’ tumor recurrence. This inhibition was accompanied by reduced expression of stem-related gene Sox2 due to induction of p27 and miR-30a. A combination of DC120 and CDDP more effectively inhibited NPC cells compared with monotherapy *in vitro* and *in vivo*. Clinical evaluation of DC120 is warranted.

## INTRODUCTION

Nasopharyngeal carcinoma (NPC) is the most frequent head and neck tumor in South China with the highest incidence rate of up to 54.7/100,000/year [1]. Despite improvements in conventional treatment, approximately 30% of patients with locoregionally advanced disease will subsequently relapse, frequently with associated metastases, and develop resistance to first-line drugs [2]. Accumulating evidence suggests that side population cells [3], a rare population of cells from primary tumors or cancer cell lines, are enriched in a subset of tumor-initiating or cancer stem-like cells (CSLCs) [4], which contribute to tumor metastasis and recurrence as well as tumor resistance to both radiation and chemotherapy [5].

It is well established that the PI3K/AKT signaling pathway is involved in a wide variety of biological processes, including cell proliferation, differentiation, apoptosis, autophagy, glucose metabolism, DNA double-strand break repair and tumorigenesis [6–10]. Loss or mutation of tumor suppressor PTEN, amplification or mutation of PI3K, activation or mutation of growth factor receptors and oncogenes, and amplification of AKT itself are involved in the activation of AKT in tumors [10–12]. Recently, increasing evidence suggests that the activation of AKT has a key role in the function of CSLCs and modulates the percent of SP cells in a variety of cancers, including esophageal carcinoma [13], glioma tumors [14], lung cancer [15] and breast cancer [16]. Further studies demonstrate that constitutive activation of AKT promotes CSLC resistance to treatment with chemotherapy and/or radiation therapy partially by downregulating the expression of p27 [17], a well-known tumor suppressor.

AKT kinase has become an attractive target for small molecular drug discovery. To date, researchers have developed many AKT inhibitors, including targeting the ATP binding site, pleckstrin homology domain [18], or protein substrate binding site of AKT. Several of these inhibitors, such as MK-2206, GDC0068, and perifosine are currently in phase I and II trials alone or in combination to treat multiple types of cancers [3, 19]. To find compounds that target AKT kinase, we designed and synthesized a series of 2-pyrimidyl-5-amidothiazole compounds based on the ATP binding site of AKT. We had previously reported that DC120, which was screened out from these compounds, exhibited an inhibitory effect on cancer cells *in vitro* and *in vivo* through the inhibition of AKT kinase activity and the blockade of the AKT downstream signaling pathway [20, 21]. In this study, we sought to explore the involvement of DC120 in the regulation of NPC cancer stem-like SP cells. Our data showed that DC120 inhibited the proliferation of human NPC CNE-2-S-18/SP and CNE-1/SP cells *in vitro* and *in vivo* and significantly reduced the self-renewal and tumor-initiating capacities of cancer stem-like SP cells via the induction of cell apoptosis. Additionally, we observed that DC120 suppressed the cancer stem-like SP cells through the inhibition of AKT kinase activity and the blockade of the PI3K/AKT downstream signaling pathway, further regulating Sox2 expression. Moreover, we found that the combination of DC120 and cisplatin (CDDP) has a significant synergistic effect, and DC120 could sensitize the inhibitory effect of CDDP on NPC cells.

## RESULTS

### NPC SP cells have the characteristics of cancer stem-like cells (CSLCs)

**Figure 1:**
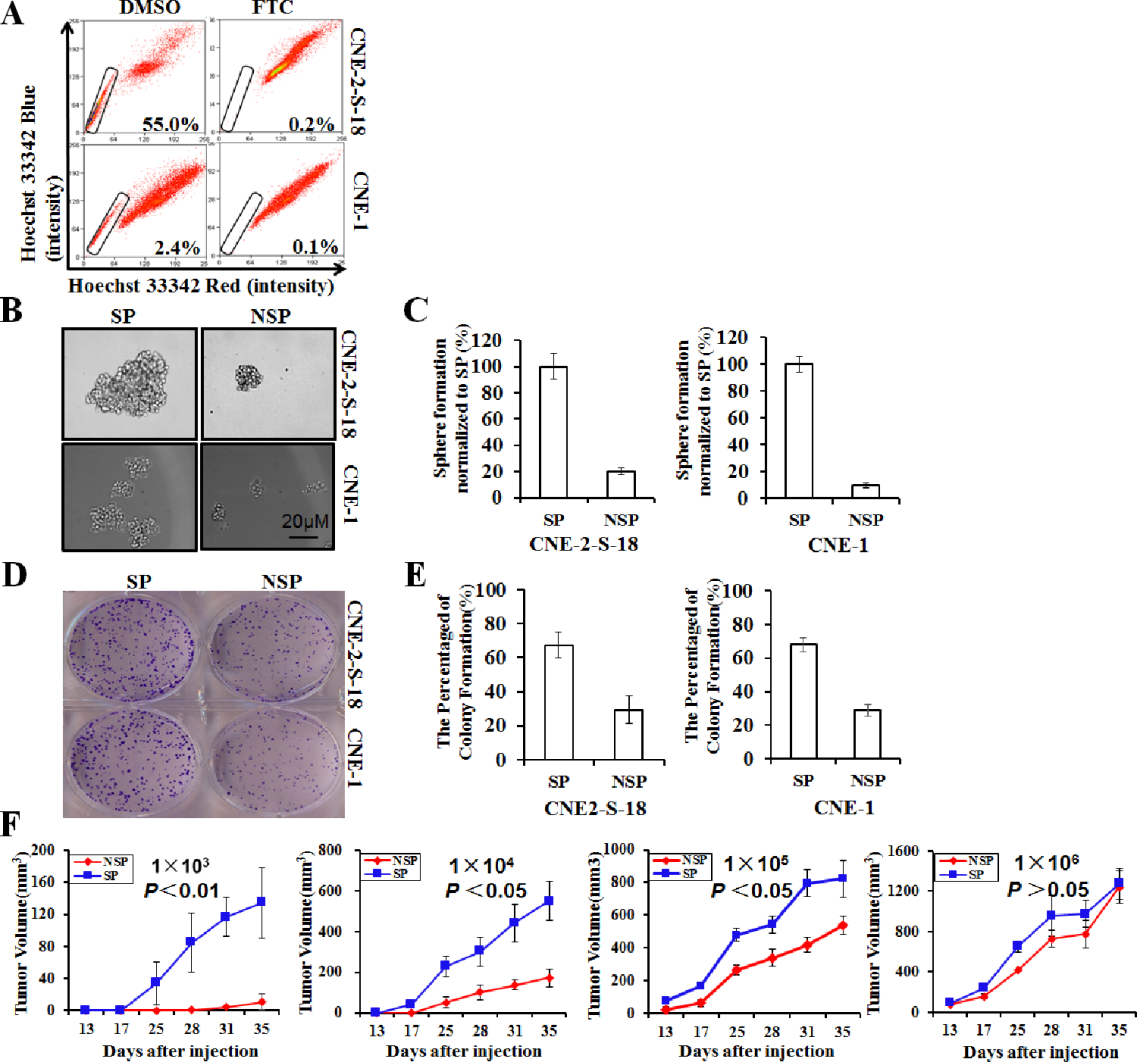
Identification and characterization of cancer stem-like SP cells in NPC cell lines **(A)** Human NPC lines CNE-2-S-18 and CNE-1 were labeled with Hoechst 33342 dye and analyzed by flow cytometry with or without treatment with Fumitremorgin C (FTC). **(B–C)** SP and NSP cells were cultured in sphere-forming conditions for 7 days, counted and photographed at the same magnification. The size of the spheres was estimated using V = (4/3) πR^3^. Magnification, 100 ×. Columns, mean (*n* = 3); bars, SD. The number of spheres greater than 50 cells was counting. **(D–E)** SP and NSP cells were plated in triplicate at 200 cells per well in 6-well plates, and cultured for approximately 7 days. Colony Formation Efficiency estimated using CFE = the number of colony forming/200 × 100%. Columns, mean (*n* = 3); bars, SD. The number of colony greater than 50 cells was counting. **(F)** Tumor growth curves after injection of NOD/SCID mice with the limited dilution concentration of CNE-2-S-18/SP or CNE-2-S-18/NSP. Once they became palpable, the CNE-2-S-18/SP tumor (red) cells grew at a higher rate than the CNE-2-S-18/NSP (blue) cells in all cases.

It is believed that certain ATP-binding cassette (ABC) transporters (e.g. ABCG2/BCRP) can pump out the fluorescent dye Hoechst 33342, which may be why the SP phenotype exhibits a low level of Hoechst fluorescence intensity [14]. Using a FACS assay, we sorted SP cells in human NPC cell lines CNE-2-S-18 and CNE-1, which were characterized by a low fluorescent “tail” in the flow cytometry histogram (Figure 1A). In the present study, we found that the average percent of SP cells was approximately 60.0% in the CNE-2-S-18 cell line and approximately 2.0% in the CNE-1 cell line; which was consistent with the results of previous studies [22], however, 5 μM FTC, the ABCG2-specific inhibitor, could significantly decrease the SP proportion to 0.2% (*P* < 0.01) and 0.1% (*P* < 0.01), respectively. We also examined whether SP cells sorted through a FACS assay displayed abilities associated with human CSLCs. We observed that not only the size of the spheres increased by 8- to 125-fold (*P* < 0.01; Figure 1B), but also the number of spheres of SP cells increased by approximately 5-fold (*P* < 0.01; Figure 1C) relative to matched NSP cells when grown in suspension cultures, an *in vitro* measure of CSLC self-renewal activity. The result of colony formation assay indicated that SP cell proliferation were better than that of NSP cell (Figure 1D and 1E). We next directly estimated the tumor-initiating capacity by injecting sorted CNE-2-S-18/SP cells and CNE-2-S-18/NSP cells into NOD/SCID mice. Tumors were generated with 1,000 SP cells, which was 10-fold less than was required for tumor seeding by NSP cells and grew at a faster rate compared with CNE-2-S-18/NSP cells (Figure 1F, Table 1).

**Table 1:** Tumor-initiating capacity of CNE-2-S-18/SP cells and CNE-2-S-18/NSP cells in NOD/SCID mice

NO.	Cellname	NO.tumor/NO.injectios					
		Days after injections					
		13	17	25	28	31	35
1 × 10^3^	NSP	0/8	0/8	0/8	0/8	1/8	2/8
	SP	0/8	0/8	3/8	5/8	7/8	8/8
1 × 10^4^	NSP	0/8	0/8	3/8	3/8	6/8	6/8
	SP	0/8	3/8	8/8	8/8	8/8	8/8
1 × 10^5^	NSP	1/8	1/8	4/8	4/8	7/8	8/8
	SP	4/8	6/8	8/8	8/8	8/8	8/8
1 × 10^6^	NSP	7/8	8/8	8/8	8/8	8/8	8/8
	SP	8/8	8/8	8/8	8/8	8/8	8/8

### DC120 down-regulated the activated PI3K/AKT pathway in NPC cancer stem-like SP cells

**Figure 2:**
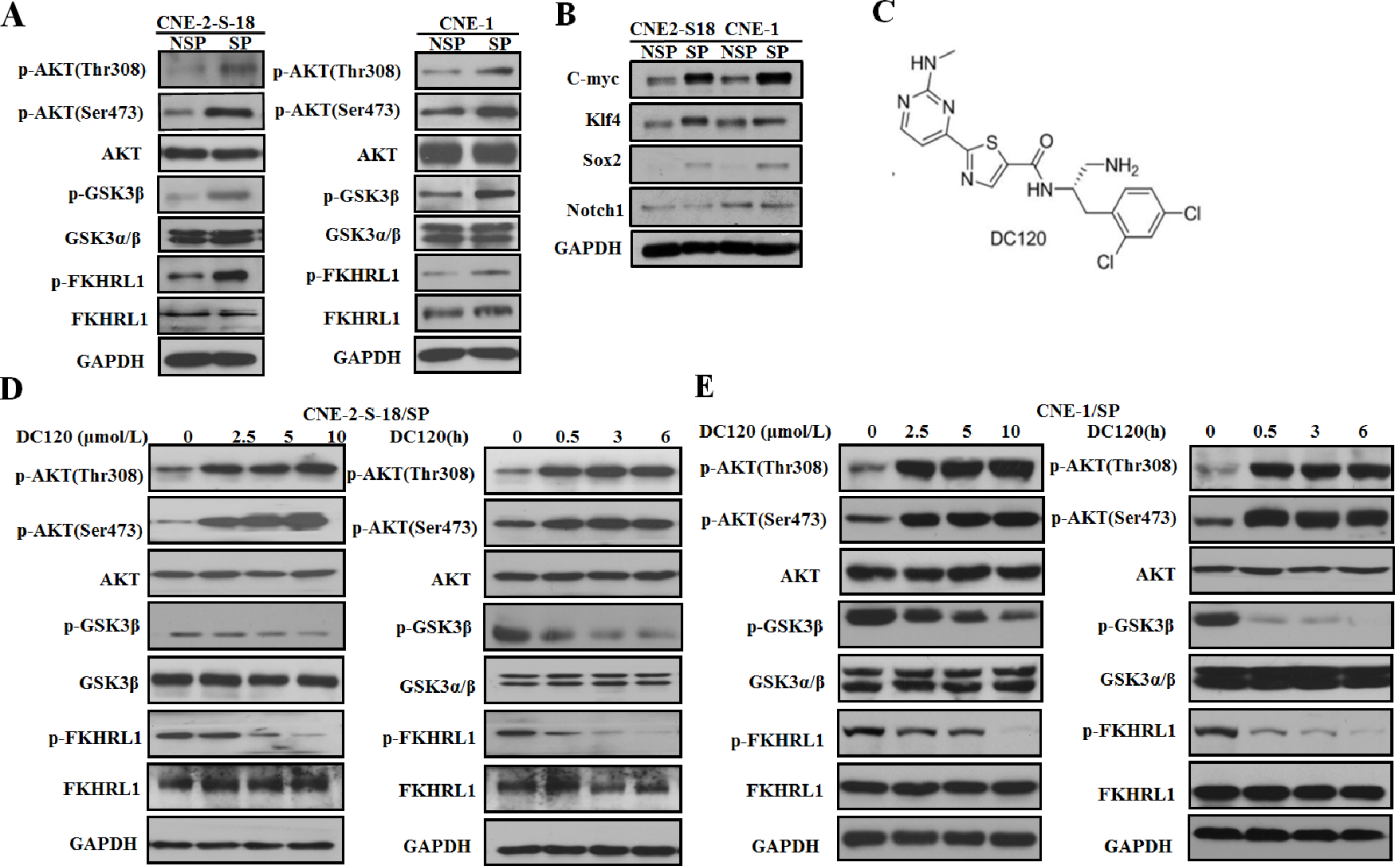
The effect of DC120 on phosphorylation of AKT downstream targets in NPC cancer stem-like SP cells **(A)** The expression levels of AKT kinase and its downstream targets of freshly sorted SP and NSP cells were analyzed by immunoblotting. **(B)** The expression levels of stem cell transcription factors of freshly sorted SP and NSP cells were analyzed by immunoblotting. **(C)** The chemical structure of DC120. **(D–E)** Freshly sorted SP cells of CNE-2-S-18 and CNE-1 cells were treated with different concentrations of DC120 for 24 h or 10 μmol/L DC120 for various amounts of times. Total isolated protein was analyzed by immunoblotting with the indicated antibodies. The results are representative of three different experiments. Control: 0.1% DMSO.

As reported, the activation of the PI3K/AKT pathway plays an important role in the maintenance of cancer stem-like SP cells [4, 23]. Among the cancer cell lines used in this study, both CNE2-S18 and CNE1 cell lines were previously confirmed to have hyper-activated PI3K/AKT signaling due to the PIK3CA and HRAS mutation, respectively. Our results indicated that the phosphorylation status of AKT on Thr308 and Ser473 and the phosphorylation levels of AKT downstream targets (FKHRL1 and GSK-3β) were much higher in SP cells than those in NSP cells (Figure 2A), suggesting that the PI3K/AKT pathway was activated in NPC cancer stem-like SP cells. We also verified the expression of stem cell transcription factors in SP and NSP cells, and found that the expressions of C-myc, klf4, Sox2 were higher in SP than in NSP, which further confirmed that the SP cells has the characteristics of stem cells (Figure 2B). As the inhibition of substrate phosphorylation can reflect the inhibition of AKT activity, we examined whether DC120 (Figure 2C) could inhibit AKT and its downstream targets. Figure 2D and 2E showed that the phosphorylation levels of FKHRL1 and GSK-3β were all partially attenuated by DC120 dose and time dependently without affecting the amount of total proteins. However, the phosphorylation of Thr308 and Ser473 on AKT increased concomitantly, although AKT kinase activity was inhibited, the conformational change of AKT led to its self-hyperphosphorylation. More precisely, phosphorylation of FKHRL1 and GSK-3β was reduced within 30 minutes after exposure to 10 μmol/L DC120 in CNE-2-S-18/SP and CNE-1/SP cells. These data suggested that the down regulation of the PI3K/AKT self-renewal pathway might contribute to the inhibitory effects of DC120 on NPC cancer stem-like SP cells.

### DC120 inhibited NPC cancer stem-like SP cells *in vitro*

**Figure 3:**
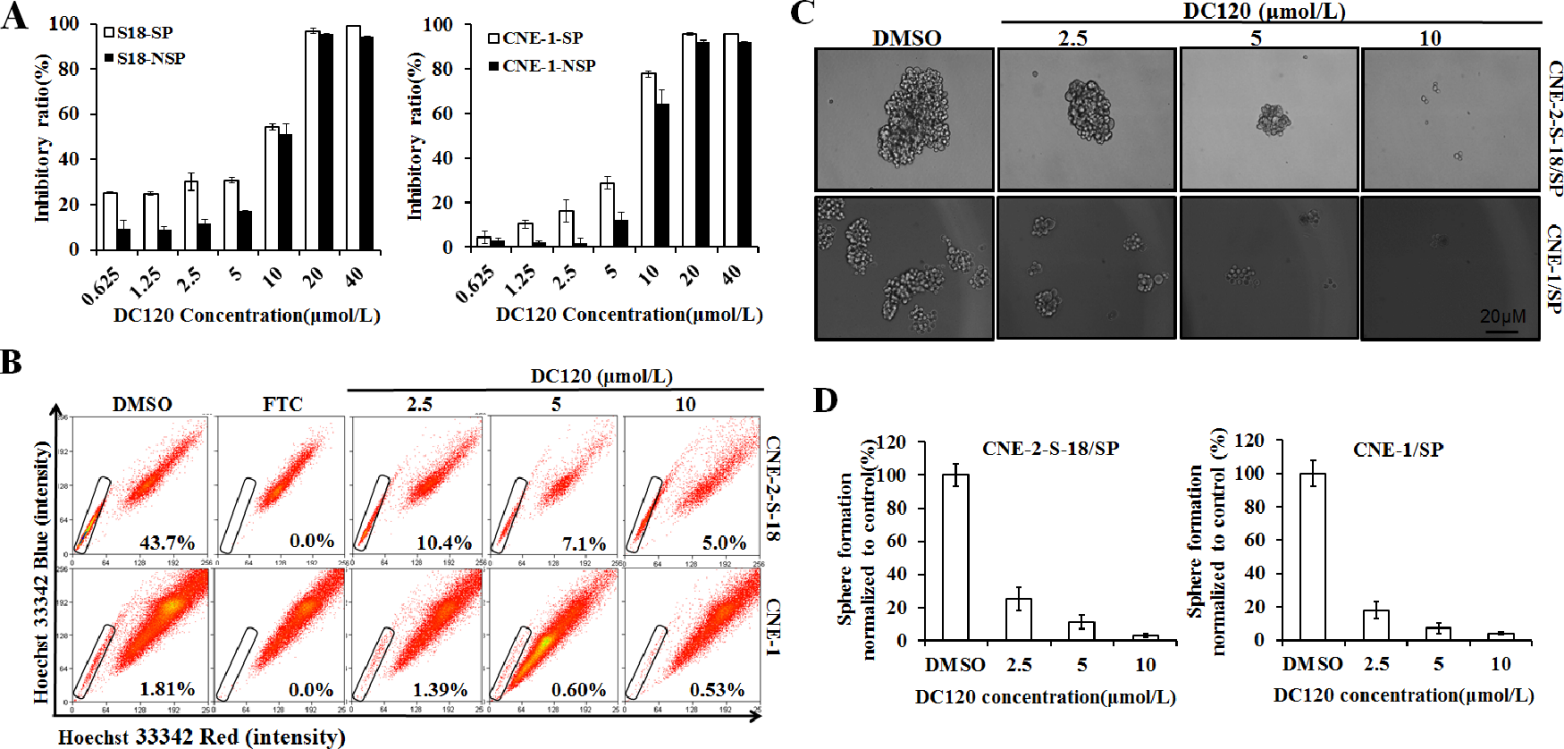
Inhibitory effect of DC120 on cancer stem-like SP cells **(A)** Freshly sorted SP and NSP cells of CNE-2-S-18 and CNE-1 cells were treated with increasing concentrations of DC120 for 48 hours. The antiproliferative effect of DC120 was measured by MTT assay. **(B)** Cells were treated with DC120 (2.5–10 μmol/L) for 24 hours, then labeled with Hoechst 33342 dye and analyzed by flow cytometry. A set of representative flow cytometry dot plots is shown. **(C–D)** Sorted SP cells were cultured in nasosphere-forming conditions and incubated with DC120 (2.5–10 μmol/L) or DMSO for 7 days. The size of the nasospheres was estimated using V = (4/3) πR^3^. Magnification, 100 ×. Columns, mean (*n* = 3); bars, SD.

Using an MTT assay, we determined the effect of DC120 on the proliferation of human NPC SP and NSP cells. S-18-SP/NSP and CNE-1 SP/NSP cells were sorted by FACS analysis assay and then exposed to DC120 (0.625–40 μmol/L) for 48 hours. As shown in Figure 3A, exposure to DC120 resulted in a dose-dependent inhibition of cell viability, and compared with NSP cells, SP populations were more sensitive to DC120 especially at low doses for S-18 and CNE2 cells.

To examine whether DC120 could inhibit the SP phenotype *in vitro*, we performed a FACS analysis assay. As shown in Figure 3B, 2.5 μmol/L DC120 significantly decreased the number of SP cells by over 76% in the CNE-2-S-18 cell line (*P* < 0.01) and 23% in the CNE-1 cell line (*P* < 0.05), and 10 μmol/L produced a greater than 89% reduction of SP cells in the CNE-2-S-18 cell line (*P* < 0.01) and 71% in the CNE-1 cell line (*P* < 0.01). To evaluate whether DC120 could suppress the formation of nasospheres *in vitro*, we exposed freshly sorted CNE-2-S-18/SP and CNE-1/SP cells to varying concentrations of DC120 and then cultured them for 7–14 days in the presence of the compound. As shown in Figures 3C and 3D, DC120 inhibited the formation of spheres. Not only did the number of spheres decline by 75% to 99% (*P* < 0.01; Figure 3D) but also the size of the spheres was reduced by 8- to 132-fold (*P* < 0.01; Figure 3C). The IC_50_ values were approximately 0.5–1 μmol/L for both the CNE-2-S-18/SP and CNE-1/SP spheres. Another recognized AKT inhibitor GDC0068 was employed, the same effect was obtained (Figure S1A–S1C). These data showed that DC120 inhibited the cancer stem-like SP cells at similar concentrations to those that inhibited nasosphere formation and at approximately 7-fold lower concentrations than those that inhibited cancer cells as determined by the MTT assay.

### DC120 induced apoptosis in NPC cancer stem-like SP cells

**Figure 4:**
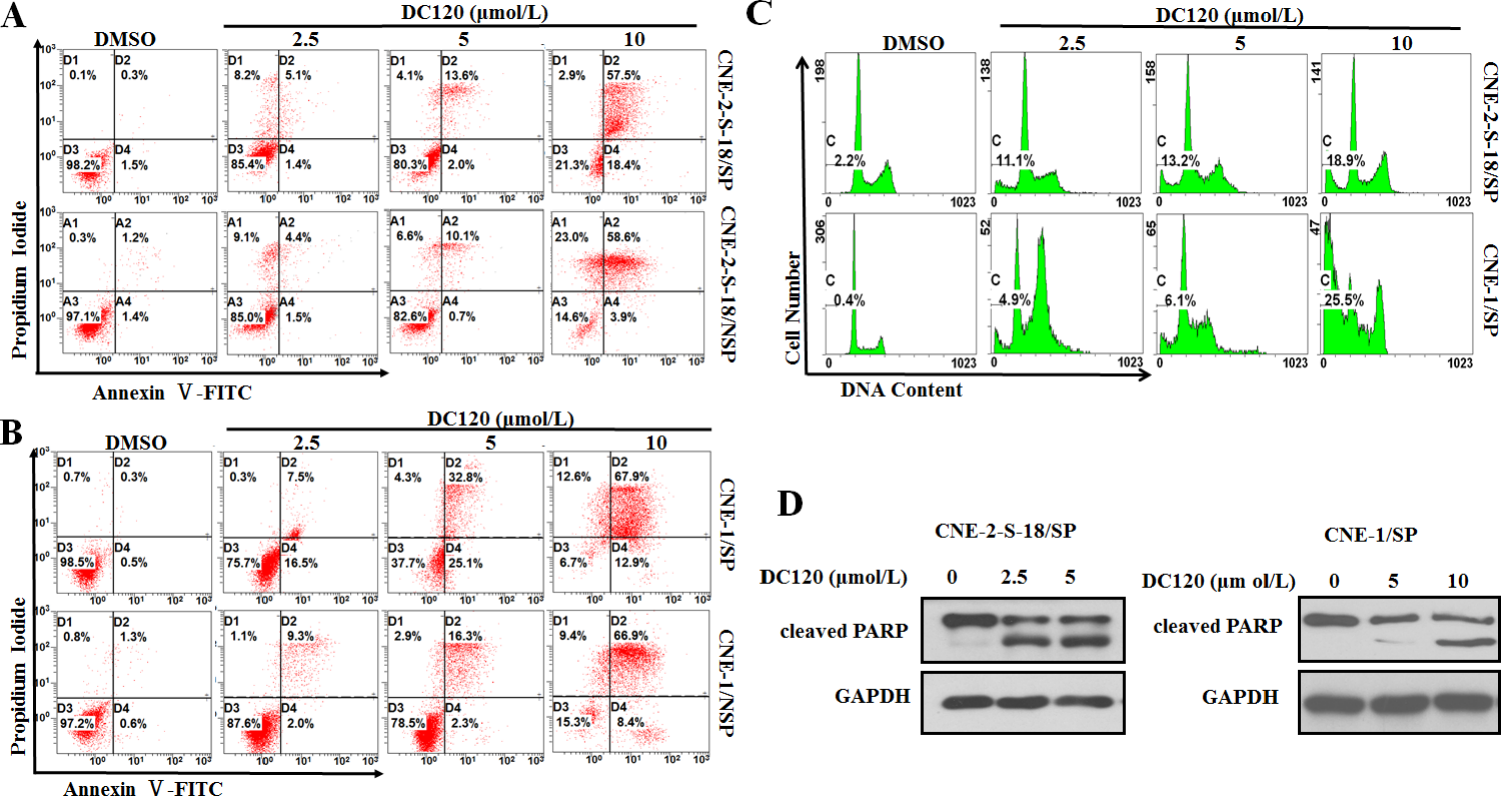
DC120 induced apoptosis in NPC cancer stem-like SP cells **(A–B)** Freshly sorted SP and NSP cells of CNE-2-S-18 and CNE-1 cells were treated with increasing concentrations of DC120 for 48 hours. The cells were harvested and stained with Annexin-V-FLUOS Solution and PI Solution, then, cells were analyzed by flow cytometry. **(C)** CNE-2-S-18/SP and CNE-1/SP cells treated with different concentrations of DC120 for 48 h. The cells were harvested and fixed overnight in 75% ethanol at −4°C and resuspended in 1 ml of PI staining solution (50 μg/ml PI, 50 μg/ml RNase) for 15 min. The PI fluorescence associated with DNA was measured by a flow cytometer. **(D)** CNE-2-S-18/SP and CNE-1/SP cells treated with different concentrations of DC120 for 48 h. Western blot analysis was conducted and probed with a cleaved PARP antibody. Control: 0.1% DMSO.

To confirm whether DC120 inhibits the cancer stem-like SP cell phenotype by inducing apoptosis *in vitro*, an Annexin V-FITC/propidium iodide double staining assay was used to detect the apoptotic cells. In Figure 4A, the percentage of Annexin V-positive cells were higher in CNE-2-S-18/SP cells (6.5%, 15.6%, 75.9%) than that in CNE-2-S-18/NSP cells (5.9%, 10.8%, 61.5%), when CNE-2-S-18-SP/NSP cells treated with 2.5, 5 or 10 μmol/L DC120 for 48 hours. When CNE-1-SP/NSP cells were treated with 2.5, 5 or 10 μmol/L DC120 for 48 hours, the percentage of Annexin V-positive cells was 24%, 57.9%, 80.8% and 11.3%, 18.6%, 75.3% respectively (Figure 4B). Furthermore, the sub-G1 fraction was tested using flow cytometric analysis to identify the apoptotic cell population. When the cells were exposed to 10 μmol/L DC120 for 48 hours, the rates of the sub-G1 fraction ranged from 2.2% to 18.9% in CNE-2-S-18/SP cells and from 0.4% to 25.5% in CNE-1/SP cells (Figure 4C). Moreover, poly (ADP-ribose) polymerase (PARP) was cleaved to yield a 110/85-kD fragmentation and was also detected in CNE-2-S-18/SP and CNE-1/SP cells following DC120 treatment (Figure 4D). These data indicated that DC120 indeed induced apoptosis in NPC SP cells, which was consistent with the results of the nasosphere-forming assay and SP analysis assays.

### DC120 effectively sensitized NPC cells to CDDP therapy *in vitro*

**Figure 5:**
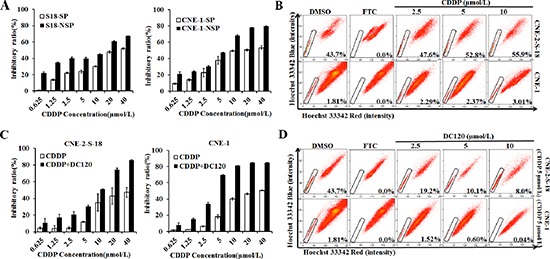
Treatment of NPC cells in combination with DC120 and CDDP *in vitro* **(A)** Freshly sorted SP and NSP cells of CNE-2-S-18 and CNE-1 cells were treated with increasing concentrations of CDDP (0.625–40 μmol/L) for 48 hours. The anti-proliferative effect of CDDP was measured by MTT assay. **(B)** Cells were treated with CDDP (2.5–10 μmol/L) for 24 hours, then labeled with Hoechst 33342 dye and analyzed by flow cytometry. A set of representative flow cytometry dot plots is shown. **(C)** Cells were treated with increasing concentrations of CDDP (0.625–40 μmol/L) and a fixed concentration of DC120 (2.5 μmol/L) for 48 hours. The anti-proliferative effect was measured by MTT assay. **(D)** Cells were treated with increasing concentrations of DC120 (2.5–10 μmol/L) and a fixed concentration of CDDP (5 μmol/L) for 24 hours. The percentage of SP cells decreased significantly.

Cisplatin (CDDP) is a clinically active agent against NPC. However, in contrast to DC120, NSP cells were more sensitive to CDDP than SP cells in CNE-2-S-18 and CNE-1 cells (Figure 5A). Moreover, CDDP treatment increased the percentage of SP cells (Figure 5B), which was similar to other findings and may play a role in resistance [24]. We further examined the combination of CDDP and DC120, which had a significant synergistic effect (Figure 5C) and produced a combination index (CI) value of < 1 in both CNE-2-S-18 and CNE-1 cells (Table 2 and Table 3). Although treatment with CDDP alone increased the proportions of cancer stem-like SP cells (Figure 5B), 5 μmol/L or 10 μmol/L DC120 plus 5 μmol/L CDDP significantly decreased the proportions of SP cells by over 76.9% (*P* < 0.01) or 81.7% (*P* < 0.01) in CNE-2-S-18 cells, and 66.9% (*P* < 0.01) or 97.8% (*P* < 0.01) in CNE-1 cells, respectively (Figure 5D).

**Table 2:** CIs generated from isoblogram at increasing concentrations of DC120 and CDDP in CNE-2-S-18 cells

DC120(μmol/L)	CDDP(μmol/L)	Fa	CI
0.625	0.625	0.107	0.334
1.25	1.25	0.173	0.418
2.5	2.5	0.204	0.704
5	5	0.304	0.901
10	10	0.508	0.881
20	20	0.745	0.753

**Table 3:** CIs generated from isoblogram at increasing concentrations of DC120 and CDDP in CNE1 cells

DC120(μmol/L)	CDDP(μmol/L)	Fa	CI
0.625	0.625	0.081	0.632
1.25	1.25	0.153	0.691
2.5	2.5	0.340	0.573
5	5	0.693	0.331
10	10	0.813	0.382
20	20	0.848	0.619

### DC120 and CDDP combination therapy effectively diminished cancer stem-like SP cells *in vivo*

**Figure 6:**
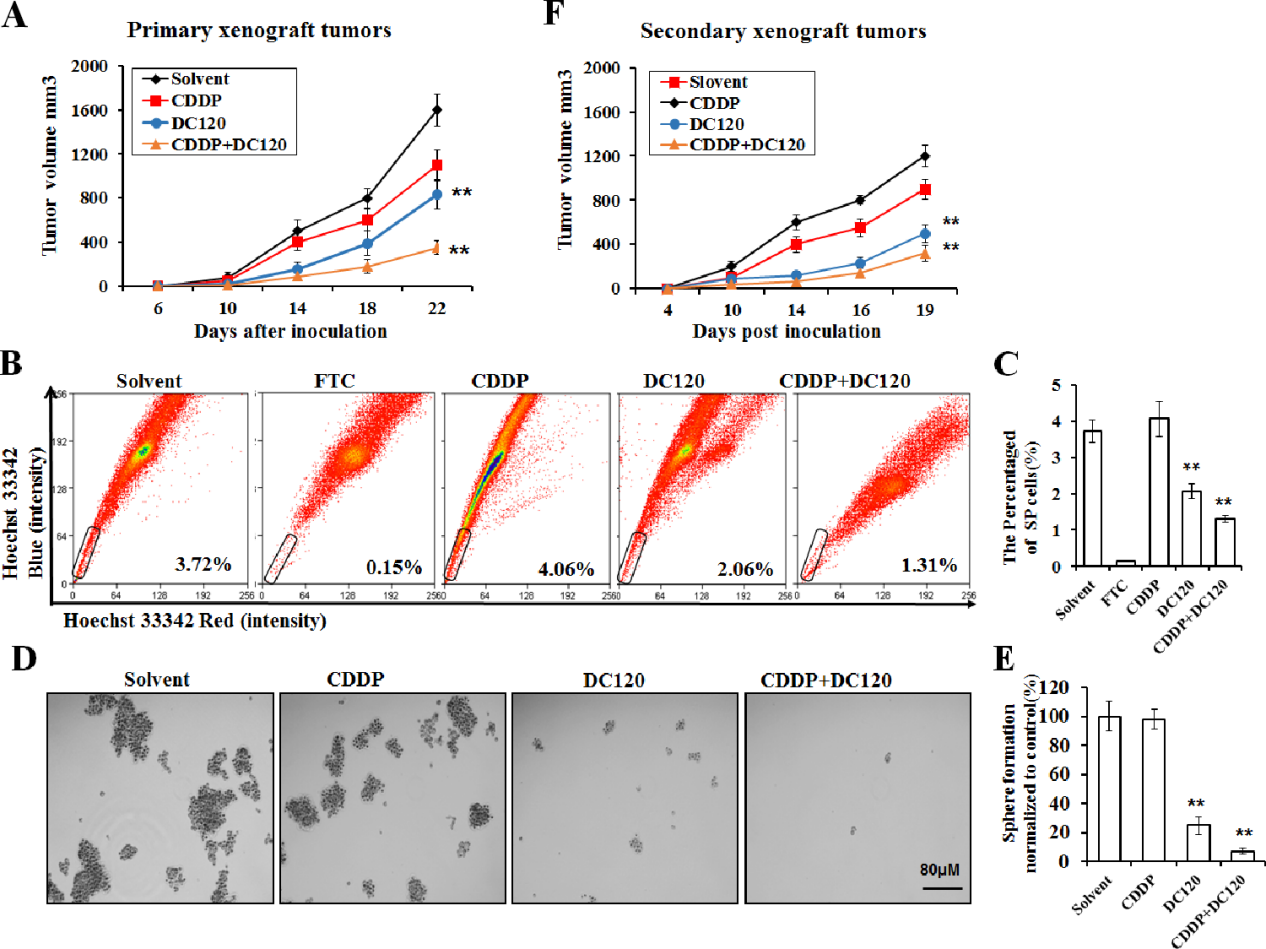
DC120 decreased tumor size and eradicated cancer stem-like SP cells *in vivo* **(A)** Mice bearing CNE-2-S-18 cells as primary xenografts were given i.p. administration of 8% solvent (vehicle group), 2.5 mg/kg CDDP every 4 days, 20 mg/kg DC120 daily, or DC120 daily + CDDP every 4 days for 15 days. The tumor volumes were determined as described in Materials and Methods. Tumor growth inhibition was calculated. **P < 0.05. **(B–C)** The tumors were isolated from the treated animals, and single tumor cells were analyzed by FACS assay, A set of representative flow cytometry dot plots is shown. **(D–E)** The tumors were isolated from the treated animals, and single tumor cells were seeded into ultra-low adhesion 6-well plates and cultured in sphere-forming conditions for approximately 7 days. **(F)** DC120 eradicated SP cells *in vivo* as assessed by reimplantation into secondary mice. Each secondary mouse received 100,000 cells from 4 groups of primary tumor cells. Columns, mean (*n* = 6); bars, SD. **P < 0.05.

To determine whether DC120 could target NPC cancer stem-like SP cells *in vivo*, we established primary CNE-2-S-18 xenografts and gave an i.p. administration of 8% solvent (negative control), DC120, CDDP, or DC120+CDDP on day 5 after implantation. Treatment with DC120 noticeably suppressed the tumor growth, and the tumor growth inhibition (T/C %) was approximately 48.7% (*P* < 0.05; Figure 6A, Table 4). Meanwhile, the inhibitory rate of the CDDP group was 31.4% (Figure 6A, Table 4). DC120 plus CDDP treatment *in vivo* led to an even greater reduction in tumor growth, and the inhibitory rate was 78.7% (*P* < 0.01; Figure 6A, Table 4). No obvious toxicity was observed in mice receiving the above treatments (Figure S2). Then, tumors were isolated from the animals, and single tumor cells were analyzed by FACS assay or seeded into ultra-low adhesion 6-well plates in serum-free media. The proportion of SP cells isolated from DC120-treated tumors demonstrated a 44.6% reduction (*P* < 0.01; Figure 6B, 6C), and the number and size of the spheres significantly decreased 5 to 10 fold and 16 to 128 fold compared with controls (*P* < 0.05; Figure 6D, 6E). However, the tumors treated with CDDP alone increased the proportion of SP cells by 9.1% (*P* < 0.05; Figure 6B, 6C), and had a similar nasosphere-forming ability compared with untreated controls (Figure 6D, 6E). Additionally, primary xenografts treated with DC120 and CDDP in combination displayed a 64.8% reduction in SP cells (*P* < 0.01; Figure 6B, 6C) and decreased nasosphere formation (Figure 6D, 6E), Furthermore, we injected 100,000 cells obtained from 4 groups of primary tumor cells into secondary recipient mice and examined the growth of the secondary tumors. We observed that cancer cells obtained from DC120-treated mice resulted in the slowest tumor recurrence, reaching a final tumor size ranging from 300 to 500 mm^3^ in the secondary mice (Figure 6F). But the cancer cells from DDP-treated mice showed the most rapid tumor regrowth, reaching a final tumor size ranging from 600 to 800 mm^3^ in the secondary mice (Figure 6F). And the tumor regrowth in DC120 combined with CDDP treatment was the slowest among the four groups (Figure 6F). These data illustrated that DC120 in combination with CDDP showed a significant synergistic effect on the inhibition of NPC cells, and DC120 effectively sensitized NPC cells to CDDP therapy *in vivo*.

**Table 4:** Inhibitory effect of compounds on growth of human CNE-2-S-18 xenografts in nude mice

Group	Weight of tumor (g)	Inhibitory rate (%)
Solvent (8%)	1.51±0.43	−
CDDP (2.5 mg/kg/q4d)	0.71±0.30	31.4%
DC120 (20 mg/kg/qd)	0.62±0.22	48.7%
CDDP (2.5 mg/kg/q4d) + DC120 (20 mg/kg/qd)	0.32±0.26	78.7%

### DC120 repressed NPC cancer stem-like SP cells through downregulating Sox2 expression

**Figure 7:**
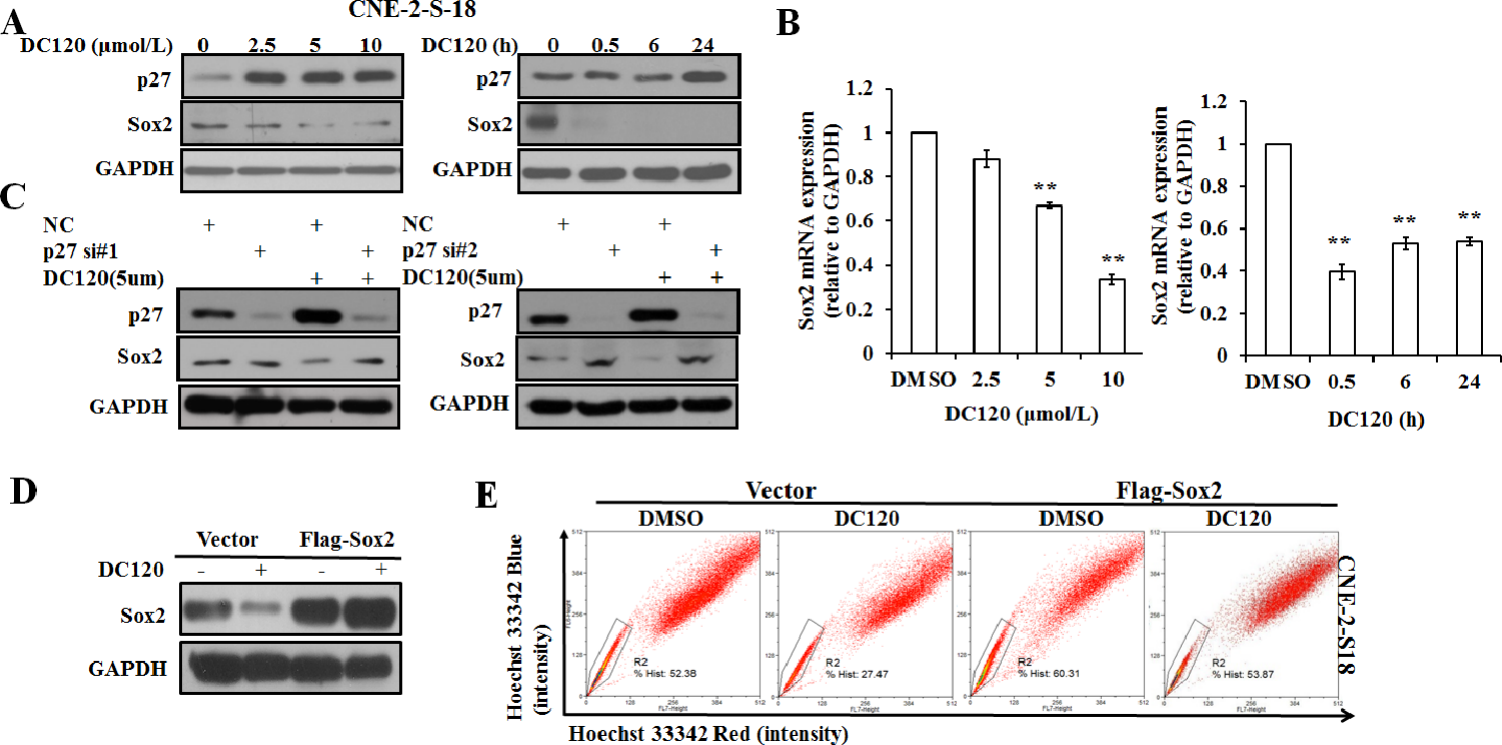
The effect of DC120 on Sox2 expression in NPC cancer stem-like SP cells **(A)** The protein expression of p27 and Sox2 following DC120 treatment. **(B)** The mRNA levels of Sox2 after treatment with DC120. **P < 0.05. **(C)** Knockdown of p27 by siRNA led to up-regulation of Sox2 in DC120-treated NPC cells. **(D)** Cells of CNE-2-S-18 were transfected with Vector or Flag-Sox2 for 24 h, then treated with DMSO or DC120 (5 μmol/L) for 24 h. The expression levels of Sox2 was tested by immunoblotting. **(E)** Cells of CNE-2-S-18 were transfected with Vector or Flag-Sox2 for 24 h, then treated with DMSO or DC120 (5 μmol/L) for 24 h. Then labeled with Hoechst 33342 dye and analyzed by FACS analysis assay. The percentage of SP cells decreased significantly.

The subsequent studies focused on the signaling pathways involved in the DC120-mediated repression of stem-like SP cells in NPC cancer cells. As mentioned earlier, as AKT can phosphorylate p27 T157 to impair the nuclear import and function of p27, constitutive activation of AKT promotes CSLC resistance to treatment with chemotherapy and/or radiation therapy partially by down-regulating the expression of p27. Inhibition of PI3K/AKT pathway by a PI3K inhibitor LY294002 or a kinase dead dominant-negative AKT mutant can up-regulate the levels of p27 in cancer cells [25, 26]. The embryonic stem cell gene SRY (sex determining region Y)-box 2 (Sox2), a transcription factor, is involved in the regulation of embryonic stem cells [24] and plays a key role in maintaining the pluripotent properties of stem cells. As a known tumor suppressor, P27 can directly repress Sox2 expression in induced pluripotent stem cells (iPSCs) through binding to the Sox2 promoter [27]. Our results showed that the protein expression level of p27 was up-regulated dose and time dependently in CNE-2-S-18 cells after treatment with DC120, while the protein expression and mRNA level of Sox2 was down-regulated dose and time dependently (Figure 7A, 7B). To further demonstrate the involvement of DC120 in the repression of Sox2 in NPC cells, two different p27 siRNAs were employed to knock down p27 in CNE-2-S-18 cells. As shown in Figure 7C, a transient transfection with 50 nM siRNA down-regulated p27 expression by at least 90%. Accordingly, the decrease of the Sox2 expression levels caused by DC120 was blocked when the expression of p27 was down-regulated. To further verified Sox2 was the target gene to regulate the efficacy of the DC120, we found overexpression of Sox2 could prevent from decrease of the SP population in NPC cells by DC120 treatment (Figure 7D, 7E). Furthermore, knockdown of the Sox2 by siRNA could decrease the proportion of SP cells in NPC (Figure S3A, S3B). Collectively, these results demonstrated that DC120 repressed Sox2 through increasing p27 expression.

Growing evidence has shown that microRNAs (miRNAs) are extensively involved in gene regulation by binding to the mRNAs of protein-coding genes to direct their posttranscriptional repression [28]. As a key regulator in development and carcinogenesis, Sox2 also displayed close associations with microRNAs. According to reports previous, at translational level, activities of Sox2 were controlled by several miRNAs, namely microRNA-145, microRNA-126, microRNA-9 and microRNA-21 [29, 30]. To explore the role of miRNAs in DC120-mediated Sox2 down-regulation, we analyzed the differences in miRNAs profiles between control (DMSO solution) and DC120 treatment in CNE-2-S-18-SP cells from miRNAs microarrays. The cells were separately cultured with a DMSO solution or 10 μmol/L DC120 for 15 h, and total RNA from all of the cells was extracted. The expressed fold changes of miRNAs from DC120-treated cells were normalized to the DMSO-treated control cells. From the results, 7616 miRNAs with significantly different expression ratios (*p* < 0.05) were selected. Among these, 80 miRNAs were up-regulated and 78 were down-regulated, date was not shown. We then chose 20 miRNAs through the qRT-PCR, and our results showed that 9 miRNAs were up-regulated after treatment with DC120, which was consistent with the results of the microarray (Figure S4A). Among these, miR-30a exhibited a high fold change (3.5-fold) according to the microarray data. Similarly, DC120-treated cells showed a higher expression of miR-30a (5.3-fold) than control cells in real-time PCR (RT-PCR) analysis (Figure S4B). These results indicated that miR-30a expression was up-regulated by DC120. To investigate the role of miR-30a in DC120-treated cells, we explored its potential targets using a bioinformatics approach of complementary base pairing (Figure S4C). The relative luciferase activity of the reporter that contained the wild-type 3′ UTR was significantly suppressed when miR-30a was cotransfected (Figure S4D). In contrast, the luciferase activity of the mutant reporter was unaffected by the cotransfection of miR-30a (Figure S4D), indicating that miR-30a suppressed Sox2 gene expression using the miR-30a-binding sequence at the 3′ UTR of the Sox2 gene. Additionally, the ectopic expression of miR-30a caused a decrease in Sox2 protein expression and mRNA levels in CNE-2-S-18 cells (Figure S4E–S4G). These data demonstrate that miR-30a may directly target the Sox2 gene via seed matches on both 3′ UTR. Sox2 was down-regulated and p27 was up-regulated when CNE-2-S-18 cells were treated with another an AKT inhibitor, GDC0068, which also induced the mRNA level of Sox2 down-regulated as well as miR-30a mRNA level up-regulated (Figure S5A–S5C). Taken together, our results demonstrate that DC120 regulates Sox2 via up-regulation of p27 and miR-30a.

## DISCUSSION

Here we report that a new compound DC120 selectively targeted NPC cancer stem-like SP cells and sensitized NPC cells to conventional chemotherapy *in vitro* and *in vivo* by blocking the AKT signaling pathway and inhibiting Sox2 expression.

Cancer stem-like cells (CSLCs) refer to a subpopulation of tumor cells with important properties, including self-renewal (by symmetric and asymmetric division), differentiation capacity, chemoresistance to standard cancer therapies, cancer invasion and metastasis [5, 31–33]. The therapy of specifically targeted cancer stem cells may be the breakthrough for tumor therapy [34, 35]. Diverse cell surface markers such as CD44 and CD133 have been used for the identification of CSCs in human tumors. So far, the markers for nasopharyngeal carcinoma stem cells is not clear. Hoechst-excluding side populations sorted from hematological and solid malignancies, including ovarian cancer [24], breast cancer [36], esophageal carcinoma [13] and nasopharyngeal carcinoma [37], have been reported to be enriched for CSLCs. In our study, we also found that NPC cancer stem-like SP cells had the ability of self-renew and initiate tumors through nasosphere formation assays, colony formation assays and NOD/SCID xenograft assays (Figure 1B and 1F).

Activation of the PI3K/AKT pathways is critical for the regulation and maintenance of cancer stem-like cells in breast cancer, prostate cancer, esophageal cancer, glioblastoma multiforme, and non-small cell lung cancer [4, 14, 15, 36, 38, 39]. Furthermore, PI3K/AKT signaling is also associated with chemoresistance in a subset of cancer cells called SP cells, which contribute to drug resistance [40]. Therefore, targeting PI3K/AKT signaling may be beneficial in cancer treatment by eliminating cancer stem-like SP cells. In this study, we first found that AKT kinase and its signaling pathway were hyper-activated in NPC cancer stem-like SP cells (Figure 2A). Additionally in the presence of DC120 phosphorylation of AKT downstream targets such as FKHRL1 and GSK-3β were markedly decreased in cancer stem-like SP cells. Simultaneously, a concomitant increase in the Thr308 and Ser473 phosphorylation of AKT was also observed (Figure 2D, 2E), which might be caused by a feedback loop induced by DC120 or a direct consequence of DC120 binding to the ATP binding site of AKT, as we previously reported [20]. Importantly, we found that DC120 could preferentially target cancer stem-like SP cells *in vitro* and *in vivo*. Firstly, we found that SP cells were more sensitive to DC120 by MTT assays (Figure 3A), then we confirmed that DC120 noticeably suppressed cancer stem-like SP cells’ self-renewal and tumor-initiating abilities by reducing the cancer stem-like SP cell fraction and nasosphere formation *in vitro* (Figure 3B and 3D). It is worth noting that the concentrations of DC120 that were capable of suppressing nasosphere formation and the proportion of cancer stem-like SP cells were much lower than those that exhibited anti-proliferative effects in CNE-2-S-18 and CNE-1 cells (Figure 3B and 3D). Another recognized AKT inhibitor GDC0068 was employed and the same effect was obtained (Figure S1A–S1C). In addition, DC120 decreased the SP percentage in xenograft tumors, and single cells isolated from these tumors lost their nasosphere-forming capacity and reduced the regrowth of tumors in secondary mice (Figure 6B and 6F), which is the more compelling evidence that DC120 is able to effectively deplete cancer stem-like SP cells *in vivo*. However, it should be noted that although DC120 preferentially targeted cancer stem-like SP cells, the compound was able to inhibit proliferation in most of the cancer cells (Figure 3A) and primary tumor growth of CNE-2-S-18 xenografts (Figure 6A). CDDP alone mainly targeted at the NSP population of NPC cells, and DC120 mostly aimed at the SP population, the combination treatment was actually more potent in suppressing NPC cell growth (Figure 5C). Further study confirmed that DC120 decreased cancer stem-like SP cell viability mainly due to induction of apoptosis, as demonstrated by an increased sub-G1 population, Annexin V-positive cells and cleaved PARP in tumor samples (Figure 4A–4D). Therefore, we suggested that DC120 could inhibit AKT kinase activity and block its signaling pathway via induction of apoptosis in NPC cancer stem-like SP cells.

The negative cell cycle regulator p27 has previously been reported as a commonly downregulated tumor suppressive protein in NPC [41]. Chan et al. [42] found that stable p27 protein expression may be due to downregulation of the p27 ubiquitination mediator Skp2 through downregulating AKT and pAKT in NPC cells. In our present study, we found that DC120 targeted AKT in cancer stem-like SP cells, and the effect was correlated with the reduction of pAKT and the increased expression of p27 (Figure 7A). Recently, a new study reported that p27 directly repressed the expression of stem cell marker Sox2 during embryonic stem cell differentiation [27]. The embryonic stem cell gene SRY (sex determining region Y)-box 2 (Sox2), a transcription factor, is involved in the regulation of embryonic stem cells [24] and plays a key role in maintaining the pluripotent properties of stem cells [43]. Further studies revealed that Sox2 was a crucial player in maintaining the stemness of GSC (Glioma stem cells) through miR-9 [44], CSCs in HNSCC (Head and Neck squamous cell carcinoma) through miR-302 [45], CSCs in breast tumors [25], mammalian neural stem cells [46], CD44+ cancer stem-like cells in EBV-associated nasopharyngeal carcinoma [47], cancer stem-cell from squamous-cell carcinoma [48] and SP (side population) cells in NSCLC [15]. All these studies revealed that Sox2 expression promoted and maintained the stemness of CSCs through similar manner.

In this study, we also proved that knockdown of the Sox2 by siRNA could decrease the proportion of SP cells in NPC (Figure S3A, S3B). Also, we found that DC120 could inhibit Sox2 expression through increasing p27 expression (Figure 7A). Significantly, Sox2 was restored when p27 was knocked down by specific siRNAs after treatment with DC120 (Figure 7C).

Apart from p27, miRNAs are one of the most common regulatory factors in NPC [49–51], and they play physiological and pathophysiological roles in cell proliferation, differentiation and apoptosis by regulating protein expression in a post-transcriptional manner [52, 53]. Wu et al. [54] identified miR-30d as a tumor suppressor, which was regulated by a new AKT/FOXO/miR-30d/MTDH signaling transduction pathway. In our study, we found that miR-30a was up-regulated after DC120 treatment (Figure S4B). Meanwhile, our results revealed that Sox2 was directly targeted by miR-30a to induce Sox2 down-regulation (Figure S4F, S4G). Above all, our study proved that DC120 inhibited Sox2 via increasing p27 and miR-30a expression. Overexpression of Sox2 prevented from decrease of the SP population in NPC cells by DC120 treatment (Figure 7D, 7E). Combined with DC120-mediated Sox2 downregulation and repression of the SP population and knockdown of Sox2-induced decrease of the SP percentage, this result suggested that DC120 could repress SP population via down-regulation of Sox2.

Combining AKT inhibitors with other cancer therapeutics is a promising way to improve the tumor therapeutic window. In this study, we found that DC120 could enhance NPC cells’ chemosensitivity to CDDP therapy. Although CDDP could inhibit cancer cell proliferation, NSP cells were more sensitive to CDDP than SP cells (Figure 5A), and it increased the percentage of cancer stem-like SP cells (Figure 5B), which further proved that conventional treatments target the bulk of cancer cells but do not affect CSCs [24]. However, DC120 effectively reversed the resistance of NPC cells to CDDP treatment (Figure 5C) and showed a synergistic effect in combination with CDDP (Table 2 and Table 3), which supports further exploration of the combination efficacy of DC120 with other anticancer agents in anticancer therapy.

In summary, our data provide evidence of DC120′s sustained anti-cancer effect in stem-like SP cells and a promising development of future therapies with DC120. DC120 preferentially suppressed the proliferation of cancer stem-like SP cells and the bulk of cancer cells. The combination of DC120 and CDDP had a synergistic effect in targeting cancer stem-like SP cells. These data provide validation for the development of DC120 to treat cancer stem-like SP cells and offer a novel treatment strategy for NPC in the future.

## MATERIALS AND METHODS

### DC120 preparation

For all of the *in vitro* studies, compound DC120 was dissolved in DMSO at a concentration of 50 mM and stored at −20°C. For the tumor xenograft studies, DC120 was formulated in 8% solvent diluent (DMSO/(Cremophor EL+ethanol), 1:3) at a concentration of 50 mg/ml. Its structure was reported previously [20, 21].

### Cell culture and reagents

The human NPC cell line CNE-1 was cultured and conserved by Sun Yat-sen University cancer center from 1982 and have been used in previous study [41], and the S18 cell line is a single cell clone of CNE-2 (a kind gift from Dr. Chao-Nan Qian in 2011, China) [55] and has also been used in previous study [22]. Both cell lines were authenticated by Applied Biosystems on Nov 16, 2012 via STR analysis. 293T cell line was obtained from the American Type Culture Collection in 2010. The cells were cultivated in DMEM medium supplemented with 10% fetal bovine serum in a 5% CO2 humidified atmosphere at 37°C. GAPDH, AKT, phospho-AKT (Ser473), phospho-AKT (Thr308), GSK3α/β, cleaved PARP, p27, Sox2, α-tubulin primary antibodies and a horseradish peroxidase-conjugated secondary antibody were purchased from Santa Cruz Biotechnology (California, CA, USA). Anti-phospho-FKHRL1, phospho-GSK3β, and chemiluminescence reagents were obtained from Cell Signaling Technology (Beverly, MA, USA). Hoechst 33342, Fumitremorgin C (FTC), DAPI, MTT and DMSO were acquired from Sigma-Aldrich (St. Louis, MO, USA).

### MTT assay

The cells were seeded in a 96-well plate (Falcon) at 3,000 to 5,000 cells per well. Cell viability was determined by MTT assay. The IC_50_ and combination index (CI) values were calculated using CalcuSyn software (Biosoft) as described previously [20, 56].

### Fluorescence-activated cell sorting (FACS) assay

The CNE-2-S-18 and CNE-1 cells or single cells obtained from primary xenograft tumors were harvested and resuspended at a concentration of 1 × 10^6^ cells/ml. Hoechst 33342 was then added to a final concentration of 5 μg/ml and incubated for 90 min at 37°C in the dark with interval mixing. After washing twice with PBS, the cells were kept at 4°C in the dark before flow cytometry analysis (EPICS ALTRA Flow Cytosorter, Beckman Coulter). Meanwhile, a subset of the cells was incubated with 5 μM FTC for 5 min at 37°C prior to adding Hoechst 33342. Flow cytometry data were analyzed using FlowJo software.

### Suspension culture

CNE-2-S-18/SP, CNE-2-S-18/NSP, CNE-1/SP and CNE-1/NSP cells or single cells obtained from primary xenograft tumors were counted, then plated at 1,000 cells per well in ultra-low attachment 6-well plates (Corning) with DMEM/F-12 medium mixed with 20  ng/ ml epidermal growth factor (BD Biosciences), 20 ng/ml basic fibroblast growth factor (Invitrogen), and B-27 supplement (Invitrogen). Then, different concentrations of DC120 were added to the SP cells, and the cells were cultured for approximately 2 weeks. The spheroids, also called nasospheres, were photographed and counted using a microscope.

### Colony formation assay

S18-SP, S18-NSP or CNE1-SP, CNE1-NSP cells were counted, plated in triplicate at 200 cells per well in 6-well plates (Corning), and cultured in DMEM (supplemented with 10% fetal bovine serum) for approximately 7 days. Then, the cells were washed twice with PBS and fixed in methanol for approximately 10 minutes. After two additional washes with PBS, the cells were dyed with crystal violet for 30 minutes. Then, the crystal violet was washed out and the numbers of the colonies were counted.

### Non-obese diabetic/severe combined immunodeficient (NOD/SCID) mouse model

The procedures involving mice and their care were in accordance with the National Institutes of Health Guide for the Care and Use of Laboratory Animals with the UKCCCR (UKCCCR, 1998). NOD/SCID mice were purchased from the animal institute of the Chinese Academy of Medical Science. CNE-2-S-18 cells were suspended in serum-free DMEM media after FACS, and both CNE-2-S-18/NSP and CNE-2-S-18/SP cells were injected subcutaneously into the left and right flank of the mice at 1 × 10^3^, 1 × 10^4^, 1 × 10^5^, and 1 × 10^6^ cells. The tumors were measured with a caliper, and the volume was calculated using V = 1/2(width^2^ × length).

### Flow cytometry

Cells were cultured in a six-well plate and exposed to DC120. The cells were harvested and stained with Annexin-V-FLUOS Solution and PI Solution provided by the Annexin-V-FLUOS Staining Kit (Roche, Switzerland) for 10–15 min or fixed overnight in 75% ethanol at −4°C and resuspended in 1 ml of staining solution (50 μg/ml PI, 50 μg/ml RNase) for 15 min. Then, apoptotic or sub-G1 phase cells were analyzed by flow cytometry (Beckman Coulter, USA).

### Western blot analysis

The cells were harvested and lysed in 1× cell lysis buffer (Cell Signaling Technology) with 1 mM phenylmethanesulfonyl fluoride (PMSF) added immediately before use. The protein concentration was estimated using a Pierce BCA protein assay kit. Equal amounts of protein (20 μg–40 μg) were separated electrophoretically on 8%–15% SDS-polyacrylamide gels, transferred onto PVDF membranes (Millipore), and analyzed as previously described [20].

### Luciferase reporter assay

A reporter assay using luciferase was performed as previously described [57]. 293T cells (2 × 10^4^) plated in a 24-well plate were cotransfected with internal control renilla luciferase (Rluc), reporter firefly luciferase (Fluc) with a wild-type or mutated Sox2 3′ UTR, and either miR-30a minics or a miR-30a inhibitor using Lipofectamine 2000 (Invitrogen) according to the manufacturer's instructions. Fluc and Rluc activities were assayed using the Dual Luciferase Reporter Assay System (Promega, Fitchburg, WI, USA) 48 hours after transfection, and Fluc activity was then normalized to Rluc activity. The Fluc-Sox2 3′ UTR construct was generated from 293T cell cDNA using 5′ CCGCTCGAGGGGCCGGACAGCGAACTGGAGG 3′ and 5′ATAAGAATGCGGCCGCTCAGTGTCCATATTTCAAAAATTTATTT 3′ primers. Each treatment was performed in triplicate and repeated in three independent experiments.

### Silencing of protein expression by siRNA transfection

siRNA against p27 and negative control siRNA were purchased from Santa Cruz Biotechnology. Cells were transfected with siRNA using Lipofectamine RNAiMAX Transfection Reagent (Invitrogen) according to the manufacturer's instructions. The final concentration of p27 siRNA was 50 nM. Protein expression was measured 48 h after transfection by western blotting.

### miRNA microarray

The freshly sorting CNE-2-S-18-SP cells were cultured in 6-well plates with either DMSO or 10 μmol/L DC120, and allowed to reach logarithmic growth phase. After 15 hours, total RNA was extracted and analyzed by miRNA microarrays (miRCURY LNA™ microRNA Array (v.18.0), KangChen Inc, shanghai, China). The expressed fold changes of miRNAs from DC120-treated cells were normalized to the DMSO-treated control cells. Microarray data have been deposited in GEO (Series accession number GSE59503).

### *In vivo* antitumor activity

BALB/c nude mice were obtained from Hunan SlacJingda Laboratory Animal Co. Ltd and were 4 to 6 weeks old. All manipulations were performed under sterile conditions. Primary tumor xenografts were established by injecting 2 × 10^6^ CNE-2-S-18 cells into mice. The mice were randomly divided into 4 groups, and each group contained 6 mice. The treatments were initiated on day 5 after inoculation, by which time the tumor volume had reached approximately 50 mm^3^. The treatments, including (i) 8% solvent diluent (negative control), (ii) CDDP × 4 days, (iii) DC120 daily or (iv) CDDP × 4 days+DC120 daily, were administered intraperitoneally for 16 days for each group. The tumor volumes and body weights of the mice were recorded. When all of the control tumors developed to more than 2,000 mm^3^, the nude mice were sacrificed. Tumor growth inhibition (T/C %), which was used to evaluate the tumor response to the drugs, was calculated using the ratio of the average tumor weight of the treated group (T) to the average tumor weight of the control group (C). Fresh cells from the dissociated primary tumors were suspended and counted. All of the 6 secondary nude mice were inoculated with 100,000 cells from each group of primary mouse tumors. The growth of the tumors was monitored, and the tumor volumes were measured twice weekly.

### Statistical analysis

Student's *t*-test was used to evaluate the statistical significance of the result at the 95% confidence level, and a *P* value less than 0.05 was considered statistically significance.

## SUPPLEMENTARY FIGURES


