# Supplementary material for: DC120, a novel AKT inhibitor, preferentially suppresses nasopharyngeal carcinoma cancer stem-like cells by downregulating Sox2
Source: Oncotarget. 2015 Feb 4;6(9):6944–58. doi: 10.18632/oncotarget.3128 (PMC4466661; doi:10.18632/oncotarget.3128)
Supplement: Supplementary file 1 [file oncotarget-06-6944-s001.pdf]

SUPPLEMENTARY FIGURES

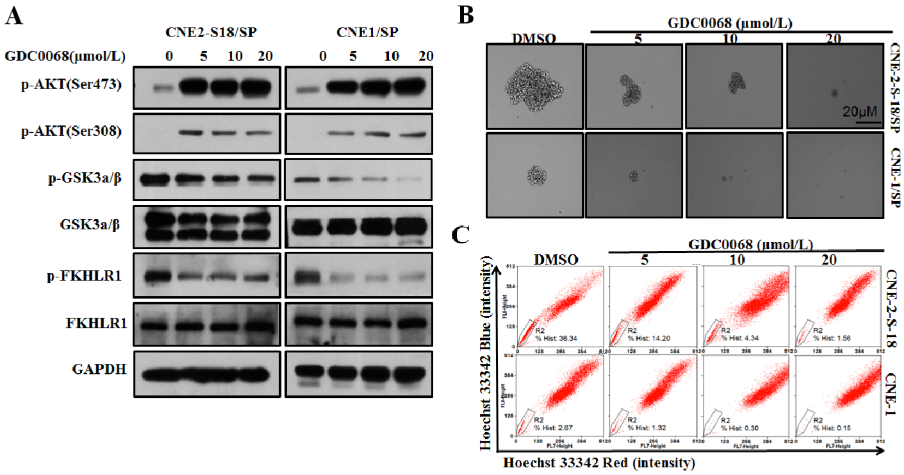

Supplementary Figure S1: Inhibitory effect of GDC0068 on cancer stem-like SP cells. (A) Freshly sorted SP cells of CNE-2-S-18 and CNE-1 were treated with different concentrations of GDC0068 (5–20 μmol/L) for 24 h. The expression levels of AKT kinase and its downstream targets were analyzed by immunoblotting. **(B)** Sorted SP cells were cultured in sphere-forming conditions and incubated with GDC0068 (5–20 μmol/L) or DMSO for 7 days. GDC0068 treatment inhibited the forming of the spheres (magnification, 100×). **(C)** Cells were treated with DC0068 (5–20 μmol/L) for 24 hours, then labeled with Hoechst 33342 dye and analyzed by FACS analysis assay. DC0068 decreased the percentage of SP cells.

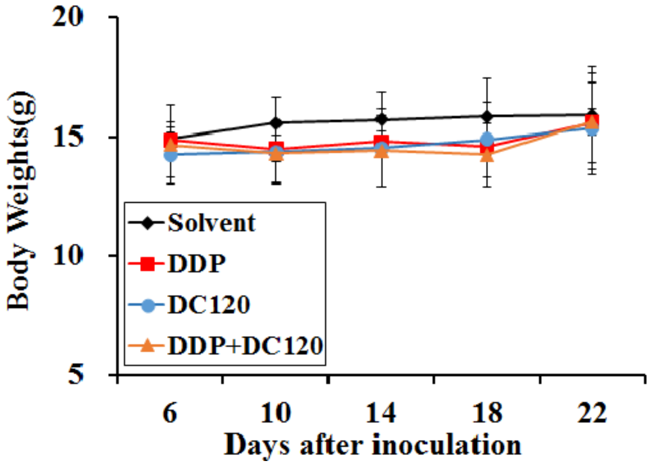

Supplementary Figure S2: Average body weights of nude mice in CNE-2-S-18 xenografts. Animals body weights was measured and recorded every 4 days during the treatment, then calculated the average value of per group.

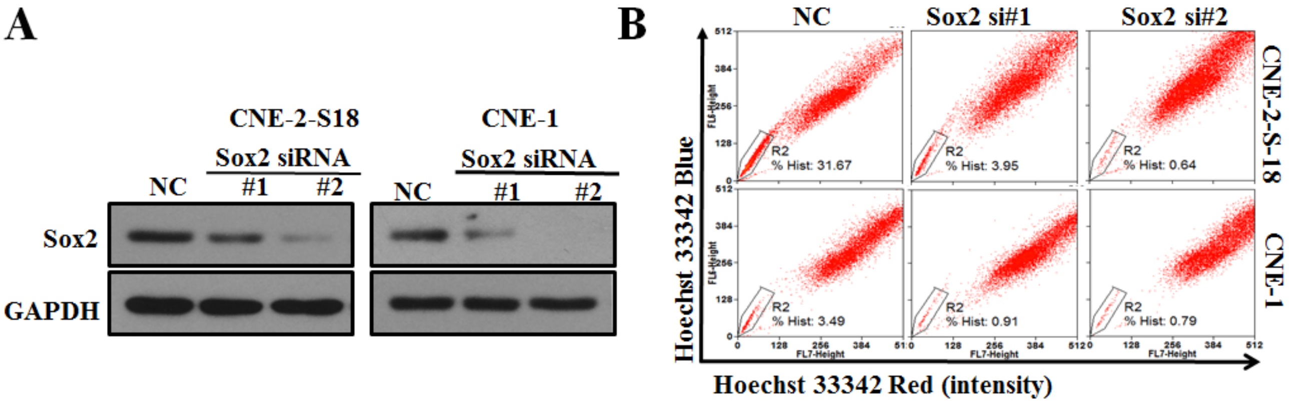

Supplementary Figure S3: Sox2 plays a key role in NPC SP cells. **(A)** Cells of CNE-2-S-18 and CNE-1 were transfected with two different siRNA of Sox2 for 48 h. The expression levels of Sox2 was tested by immunoblotting. **(B)** Cells of CNE-2-S-18 and CNE-1 were transfected with two different siRNA of Sox2 for 48 h, then labeled with Hoechst 33342 dye and analyzed by FACS analysis assay.

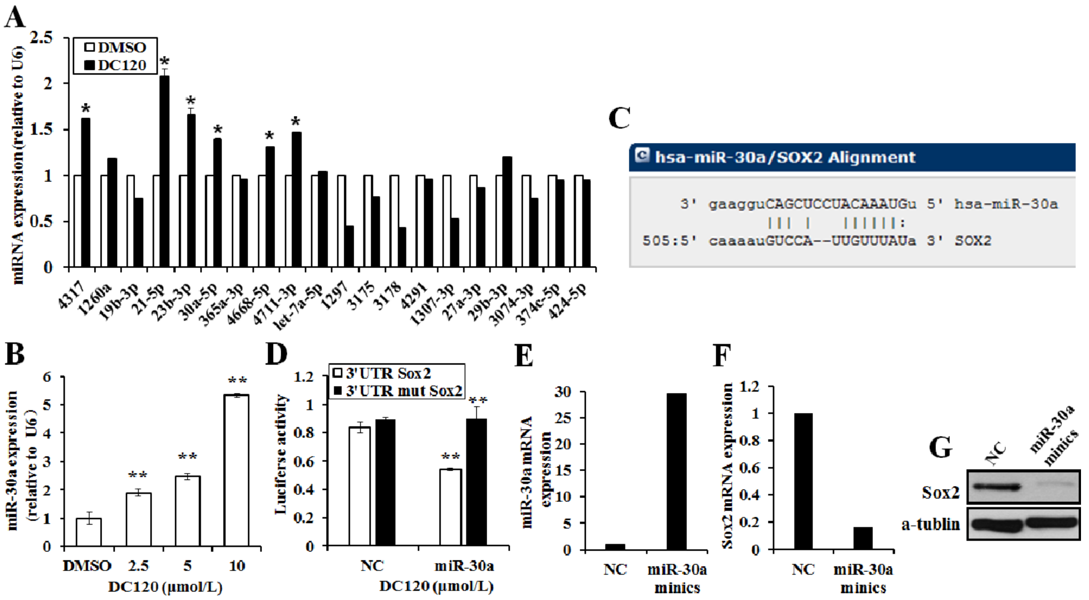

Supplementary Figure S4: MicroRNA array analysis and verification. **(A)** Cells of CNE-2-S-18 were treated with DC120 for 24 h, the mRNA levels of the indicated miRNAs were determined by qRT-PCR. **(B)** The mRNA levels of the indicated miRNAs were determined by qRT-PCR. **(C)** The sites of miR-30a binds to the Sox2 promoter which was predicted by miRanda. **(D)** Luciferase activity in 293T cells co-transfected with mimics of miR-30a or NC and Sox2 3'-UTR are shown. Data are means ± SEM; $n = 3$, $*P < 0.05$ versus NC. **(E)** The mRNA level of miR-30a in 293T cells transfected with NC or miR-30a mimics for 48 h. **(F–G)** Representative immunoblotting images and downregulation mRNA level of Sox2 in 293T cells transfected with NC or miR-30a mimics for 48 h. α-Tubulin served as an internal control.

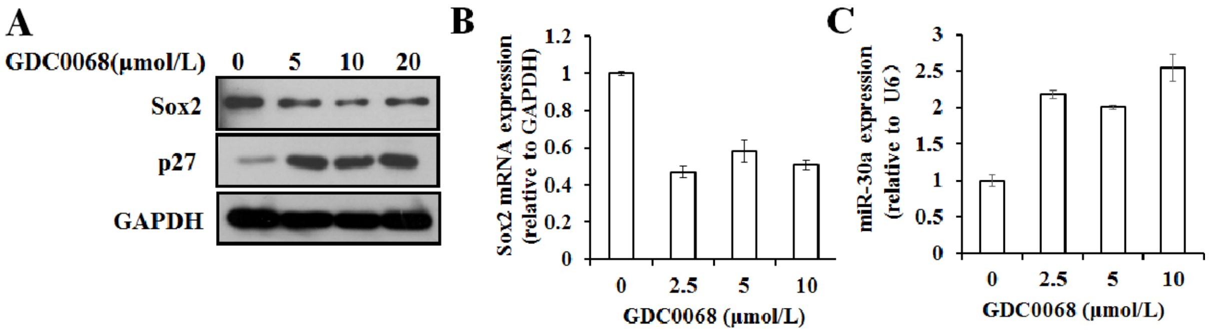

Supplementary Figure S5: The effect of GDC0068 on Sox2 expression in NPC cancer cells. **(A)** The protein expression of p27 and Sox2 following GDC0068 treatment. **(B)** The mRNA levels of Sox2 were determined by qRT-PCR after treatment with GDC0068. **(C)** The mRNA levels of the miR-30a were determined by qRT-PCR after treatment with GDC0068.
